# Secondary Tuberculosis of Breast: Case Report

**DOI:** 10.5402/2011/529368

**Published:** 2011-04-06

**Authors:** Imtiaz Wani, Ali M. Lone, Rayees Malik, Khursheed A. Wani, Rauf A. Wani, Irfan Hussain, Natasha Thakur, Vilam Snabel

**Affiliations:** ^1^Department of General Surgery, Sheri-Kashmir Institute of Medical sciences, Srinagar, Kashmir 190011, India; ^2^Department of Pathology, Sheri-Kashmir Institute of Medical sciences, Srinagar, Kashmir 190011, India; ^3^Parasitological Institute, Slovak Academy of Sciences, 04001 Košice, Slovakia

## Abstract

Tuberculosis of breast is a rare disease which is difficult to differentiate from carcinoma of breast. The involvement of breast can be primary or secondary to some focus in body. A case of secondary tuberculosis of right breast in a 21-year-old female from Kashmir, India, is being reported. Presentation was as a painless discharging sinus of right breast. A tubercular foci of rib was the affecting source of disease. No other evidence of tuberculosis was present in the body. Resection of involved rib segment, along with the discharging sinus, was performed. The patient had antitubercular therapy for 9 months, with no recurrence seen in followup.

## 1. Introduction

Breast tuberculosis is disease with rare occurrence [1]. This was first described by Sir Astley Cooper in 1829 as the “scrofulous swelling in the bosom of young women” The overall incidence in developing countries is approximately 3% of all surgically treated breath disease. High immunity conferred by breast is of significance for low recorded incidence, offering inherent resistance of breast to tuberculosis infection. Immunosuppression, development of drug-resistant strains of mycobacterium tuberculosis and global pandemic of AIDS accounted for resurgence of the disease. Breast tuberculosis commonly affects women in the reproductive age, usually between 21 and 30 years. This is seldom encountering prepubescent females and elderly women [2]. The disease may be of primary etiology when infection affects breast solely or may result from other foci in the body, which is termed as the secondary tuberculosis of breast. Presentation of breast tuberculosis is variable and may be confused with other disorders. Breast tuberculosis is often misdiagnosed as pyogenic abscess of carcinoma or breast, both clinically as well as radiologically, especially if well-defined clinical features are absent [3, 4]. Diagnosis of breast tuberculosis, therefore, remains a challenge for clinicians and requires a high degree of suspicion. Mammography or ultrasonography are unreliable in distinguishing the breast tuberculosis from carcinoma because of the variable pattern of presentation of such inflammatory lesion. An optimal radiological modality to differentiate primary tuberculosis from secondary tuberculosis of breast is computed tomography (CT) scan. Histopathology plays pivotal role in the diagnosis. Surgery is reserved only for selected refractory cases. 

## 2. Case Report

**Figure 1:**
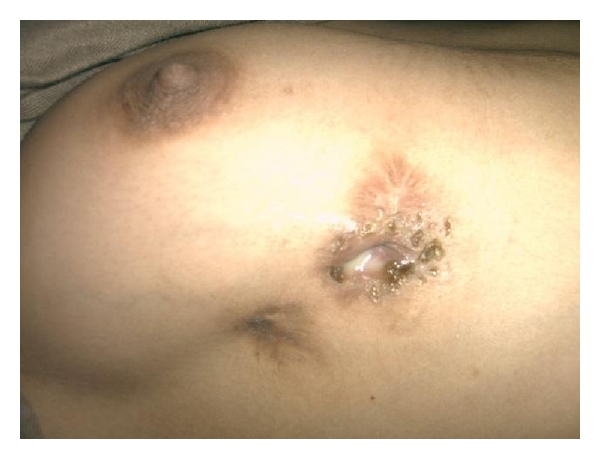
Discharging sinus present on right breast.

**Figure 2:**
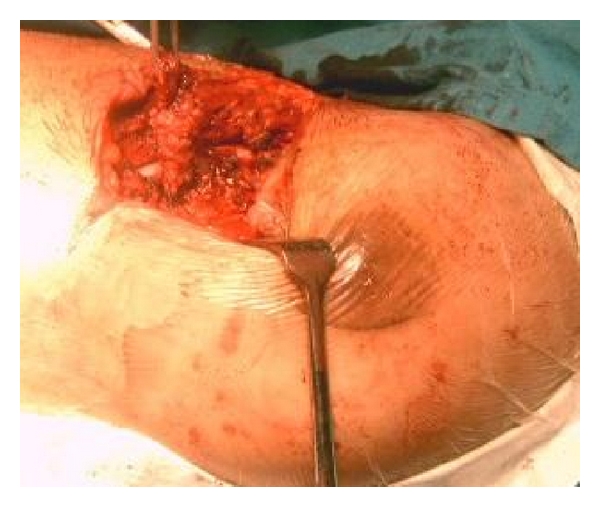
Showing necrosed tubercular rib which was foci of tuberculosis of breast.

**Figure 3:**
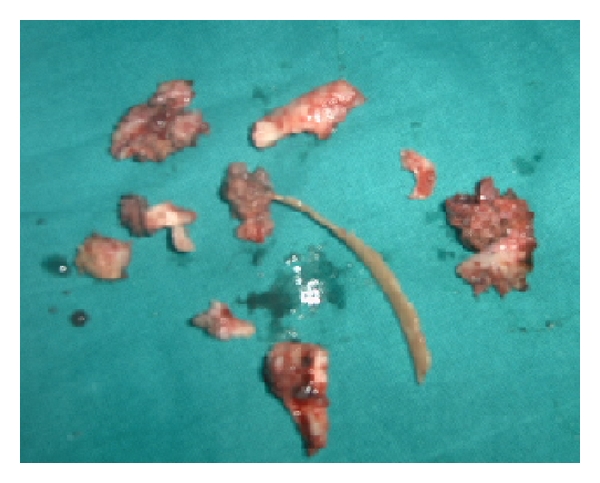
Showing resected rib with necrosed foci along with two fibrous tracts and surrounding tissue.

**Figure 4:**
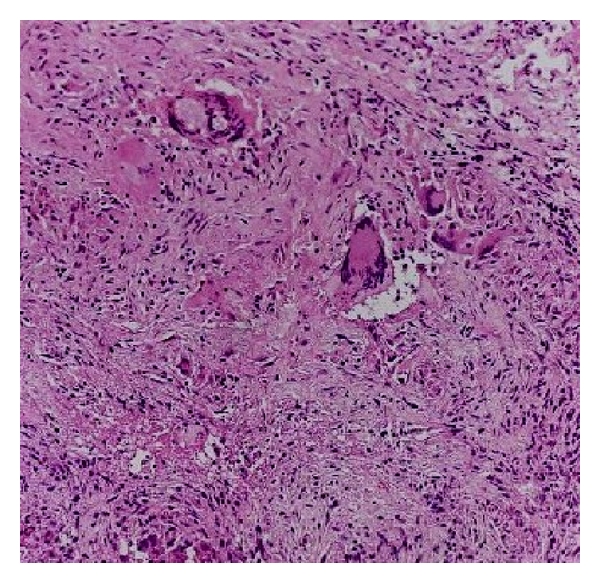
Histopathology of resected specimen showing replacement of normal tissue by mostly chronic inflammatory cell infiltrate, with areas of caseous necrosis, and giant cells are also seen.

A 21-year-old female from hilly area of Kashmir, presented with pus discharge of from two openings on the right breast for last 6 years. These aberrant openings on breast had often spontaneous pus discharge followed by cessation of discharge on its own. There were neither any constitutional symptoms nor any discharge or any abnormality of the nipple. She had already taken antitubercular therapy (ATT) thrice but was defaulter all times and reluctant for further antitubercular therapy (ATT) intake. Systemic examination was normal. Local examination showed no abnormality in left breast. A 1.5 centimeter opening was seen in inferior and outer quadrant breast, through which whitish pus was discharging along with a scar superior to discharging sinus which used to discharge pus 2 weeks before. (Figure 1). No swelling was palpable. Two firm tracts, inferior one with length of 4 cm, superior with 2.5 cm, were run posterior-superiorly ending on fifth rib. No axillary lymphadenopathy was evident. Erythrocyte sedimentation rate (ESR) was raised. Montoux test was positive (16×14 mm). X-ray chest showed an osteolytic lesion in anterior part of fifth rib. Computed tomography scan of chest could not reveal any foci of tuberculosis in lungs; only two sinus tracts in breast tissue and the necrosed rib were seen. Purulent aspirate was sterile for tubercle bacilli. Breast ultrasound showed ill-defined sinus tract and opening. Given the reluctance to antitubercular therapy (ATT) intake and recurrent disease coupled with apprehension of further followup, surgical intervention was done through fifth intercostal space. Excision of fibrous tract and resection of involved rib were carried out. (Figures 2 and 3) About 12 cm of rib containing pus and necrosed tissue were resected. Histopathology of specimen was chronic inflammatory cell infiltrate, with areas of caseous necrosis, giant cells (Figure 4). After proper counseling and understanding the nature of disease by patient and her parents the affected woman was motivated for antitubercular therapy (ATT) intake and with daily dose of 300 mg isoniazid, 600 mg rifampicin, 1500 mg pyrazinamide, ethambutol, and 10 mg pyridoxine for 9 months. She had regularly attended our followup clinics during last two years, with no recurrence of disease being recorded. 

## 3. Discussion

Breast tuberculosis is uncommon in surgical practice. Lack of awareness of manifestations and simulating benign or malignant lesions of disease are contributory to its overlooking and misdiagnosis. It may coexist with carcinoma. High incidence of breast tuberculosis is presumed in India despite only few hundred cases of breast tuberculosis reported, probably due to lack of awareness of manifestation of disease or misdiagnosis [5]. No well-defined clinical features suggestive of breast tuberculosis are defined. Involvement of breast can be unilateral or bilateral. There are only few reports documented bilateral breast tuberculosis in India [6]. Essentially, tuberculous infection is rarely confined only to the breast, and secondary tuberculosis is sequela of tuberculosis in other organs of the body. The reported incidence of isolated tuberculosis of the breast ranges from 0.10% to 0.52%. Breast tuberculosis was classified into five different types by Mckeown and Wilkinson [7]:

- nodular tubercular mastitis,
- disseminated or confluent tubercular mastitis,
- sclerosing tubercular mastitis,
- tuberculous mastitis obliterans,
- acute miliary tubercular mastitis.

Risk factors are multiparity, lactation,trauma, past history of suppurative mastitis, and AIDS. Various routes of infection described, including haematogenous, lymphatic, direct inoculation, and/or ductal infection. Occasionally, direct extension from contiguous structures such as infected rib, costochondral cartilage, sternum, shoulder joint, through the chest wall from a tuberculous pleurisy, or via abrasions in the skin can pass infection into breast [8]. A rare form of disease, disseminated form of breast tuberculosis, is characterized by multiple tubercular foci throughout the breast, which may undergo caseation leading to sinus formation. 

Tuberculosis involving ribs and presenting with breast mass is a very rare entity, and only a few cases have been to date reported in the literature. A direct fistulous tract with the pleura or a destroyed rib fragment in the abscess can be seen due to disease [9]. Symptoms in breast tuberculosis are usually seen for less than one year; its duration varied from few months to several years. Breast tuberculosis can be presented as a lump, ulcer, and breast abscess with or without discharging sinuses. Lump is commonly seen in the central or upper outer quadrant of the breast, an extension of tuberculosis from axillary nodes to the breast. Formation of fistulas and sinus tracts is usually seen in advanced disease or after needle puncture [10].

If needed, X-ray chest is done to detect any evidence of active or stigmata of healed lesion in the lungs, and Mantoux skin test positivity in adults has relevance in nonendemic areas. Fine-needle aspiration cytology aid in diagnosis of breast lumps with or without lymphadenopathy. Cytological and microbiological studies can be employed with ultrasound guided fine-needle aspiration. Diagnosis is based on identification of typical histological features or the tubercle bacilli under microscopy or culture [11]. The culture used to be positive only in 25%–30% cases. Magnetic resonance imaging may be useful in demonstrating the extramammary extent of disease. Cytological and bacteriological study of aspirate proves definitive diagnosis; this can be complemented with biopsy. In mammography, three different patterns are recognized—The nodular pattern, The disseminated pattern and the sclerosing pattern. This is difficult to differentiate between tuberculosis lesion and carcinoma on mammogram. Ultrasonography reveals heterogeneous, hypoechoic, fluid containing masses with internally floating, and echogenic material in the breast parenchyma or retromammary region in tuberculosis breast. Microscopy shows fibrous and lymphoid tissue infiltrated by large epithelioid granulomas with central acellular necrosis and many giant cells. Antitubercular therapy (ATT) with or without minimal surgical intervention forms the mainstay of treatment. Surgical intervention is required for aspiration of abscesses and excision of sinuses and masses. In resistant cases, simple mastectomy can be performed [12]. 

## 4. Conclusion

Secondary tuberculosis of breast is rare. An offending agent could be underlying tuberculosis of rib and could manifest as discharging sinus of breast. Surgery with antitubercular therapy (ATT) is treatment in breast tuberculosis secondary to rib tuberculosis.
